# Cdc42: A Novel Regulator of Insulin Secretion and Diabetes-Associated Diseases

**DOI:** 10.3390/ijms20010179

**Published:** 2019-01-06

**Authors:** Qi-Yuan Huang, Xing-Ning Lai, Xian-Ling Qian, Lin-Chen Lv, Jun Li, Jing Duan, Xing-Hua Xiao, Li-Xia Xiong

**Affiliations:** Department of Pathophysiology, Medical College, Nanchang University, Jiangxi Province Key Laboratory of Tumor Pathogenesis and Molecular Pathology, 461 Bayi Road, Nanchang 330006, China; 6301616056@email.ncu.edu.cn (Q.-Y.H.); laixingning99@outlook.com (X.-N.L.); 6302615051@email.ncu.edu.cn (X.-L.Q.); l.lyu@se15.qmul.ac.uk (L.-C.L.); lj012729@163.com (J.L.); qbdlxx@163.com (J.D.); 18160790386@163.com (X.-H.X.)

**Keywords:** Cdc42, diabetes, insulin resistance, diabetic nephropathy, cancer

## Abstract

Cdc42, a member of the Rho GTPases family, is involved in the regulation of several cellular functions including cell cycle progression, survival, transcription, actin cytoskeleton organization and membrane trafficking. Diabetes is a chronic and metabolic disease, characterized as glycometabolism disorder induced by insulin deficiency related to β cell dysfunction and peripheral insulin resistance (IR). Diabetes could cause many complications including diabetic nephropathy (DN), diabetic retinopathy and diabetic foot. Furthermore, hyperglycemia can promote tumor progression and increase the risk of malignant cancers. In this review, we summarized the regulation of Cdc42 in insulin secretion and diabetes-associated diseases. Organized researches indicate that Cdc42 is a crucial member during the progression of diabetes, and Cdc42 not only participates in the process of insulin synthesis but also regulates the insulin granule mobilization and cell membrane exocytosis via activating a series of downstream factors. Besides, several studies have demonstrated Cdc42 as participating in the pathogenesis of IR and DN and even contributing to promote cancer cell proliferation, survival, invasion, migration, and metastasis under hyperglycemia. Through the current review, we hope to cast light on the mechanism of Cdc42 in diabetes and associated diseases and provide new ideas for clinical diagnosis, treatment, and prevention.

## 1. Introduction

Diabetes mellitus (DM) has become a worldwide public health challenge with rapidly increasing rates of morbidity and mortality and attracts more and more attention in this century [1]. Moreover, diabetes has become the third major chronic disease after cancer and cardiovascular diseases [2]. The American Diabetes Association (ADA) defines diabetes as a complex and chronic disease which requires continuous medical care with multifactorial risk-reduction strategies beyond glycemic control [3]. The incidence of diabetes increases among adults aged 20 to 79, with approximately 415 million people diagnosed in 2015 and, an expected increase of 642 million by 2040 [4]. Besides, according to the latest report of diabetes alliance, China has become the largest country of diabetes [5]. In 1936, scientists divided diabetes into two groups: type 1 diabetes mellitus (T1DM), type 2 diabetes mellitus (T2DM) [6]. T1DM patients cannot produce sufficient insulin, and they subsequently undergo high blood glucose levels. T2DM is caused by genetic, environmental, behavioural and other risk factors and characterized by hyperglycemia, insulin resistance (IR), and relative insulin deficiency [7]. Islet β cell failure occurs under the influence of multiple factors during the development of the disease in T1DM or T2DM. Reduction of nearly 80% β cell mass is reported in T1DM, whereas in T2DM is almost 60% [8].

Cell division cycle 42 (Cdc42), a small GTPase of the Rho family, the total length is 191 amino acids, locates on chromosome 1p36.12 with molecular weight of 21.33 kDa. Cdc42 was first discovered in budding yeast. Cdc42 regulates cell polarity, actin cytoskeleton including filopodia formation and cell cycle progression [9]. Cdc42 gene is highly homologous and conservative in human and yeast. It is speculated that Cdc42 may play a fundamental role in mammalian cellular biological processes [10]. Activation and inactivation of Cdc42 are regulated by various regulatory factors. At present, scientists have discovered three kinds of factors: (a) guanine nucleotide exchange factors (GEFs), which exchange GDP-bound (inactive) into GTP-bound (active) and activate Cdc42; (b) GDP-dissociation inhibitors (GDIs), which are thought to sequester Cdc42 in the inactive-bound state; (c) GTPase activating proteins (GAPs), which transform Cdc42 into inactive-bound by raising its GTPase activity [11]. Cdc42 acts as a molecular switch cycling between inactive GDP-bound and active GTP-bound; early studies found that only Cdc42-GTP can be transported from the cytoplasm to the cell membrane, indicating that the binding of Cdc42 to the membrane is in the form of Cdc42-GTP [12]. Normal expression of Cdc42 not only plays critical roles in normal islet functions including cytoskeletal remodelling, vesicular transport and fusion, insulin secretion and β cell proliferation but also is essential for glucose-stimulated induced insulin secretion (GSIS) to occur [13]. In recent years, study showed that expression of constitutively active Cdc42 interfered with β cell delamination and differentiation leading to hyperglycemia [14]. The GSIS is composed of two phases: insulin in the first phase is released at a low level under resting conditions; while in the second phase, insulin releases at a higher level under high glucose (HG) conditions. Firstly, glucose is taken into β cells through glucose transporter 2 (GLUT2). Secondly, glucose metabolism triggers the elevated intracellular ATP and shuts down the ATP-sensitive potassium ion channel, and this leads to depolarization of cell membrane. Depolarization alerts the voltage-gated calcium channels, and insulin granules fuse with the cell membrane at the release sites, ultimately, insulin releases [15,16]. The first phase of insulin release happens in 10 min after glucose stimulation, and this phase is short and fast. On the contrary, the second phase is slow and consistent, but the speed of releasing declines gradually, for the insulin granules need to move from a reserve pool to the readily releasable pool [17]. This process relies on the actin cytoskeleton rearrangement, and Cdc42 is proved to be the upstream factor of cytoskeleton rearrangement signalling. Additionally, Cdc42 is irreplaceable in the transportation and location of insulin granules on the cell membrane. Cdc42 has been demonstrated to play a pivotal role in the second phase of insulin secretion. Disorder of Cdc42 will impair normal insulin secretion and contribute to diabetes.

The upstream of Cdc42 varies from miRNAs to proteins, with partly demonstrated pathways. For example, IGF-1R/MiR29a/Cdc42 pathway negatively regulates Cdc42 expression [18], while CysLTR/Arf/Cdc42, Yes/GDI/Cav-1/Cdc42, and GGPPS/GGPP/GGTase1/Cdc42 pathways significantly promote the activation of Cdc42 [19,20,21]. Pathways above have a great degree of interaction with the conventional GSIS, and with joint effects of triggering on Cdc42 to maintain the stable blood glucose concentration. The post-translational modification of Cdc42 regulates insulin secretion as well. The isoprenylation of Cdc42 enables it to attach to the cell membrane and thereby promotes the fusion and release of insulin granules [22]. Meanwhile, insulin secretion also relies on the methylation of carboxyl in Cdc42. Studies showed that the carboxyl methylation of Cdc42 in β cells requires endogenous GTP and is positively related to the concentration of glucose [23].

In T2DM, IR may be caused by dysfunction of β cells, and researchers consider IR as the primary cause of T2DM [24,25]. IR is a pathophysiological condition that is manifested as decreased insulin sensibility in insulin-sensitizing tissues including liver, adipose tissue, and skeletal muscle. Over the past decade, most research has emphasized that obesity is a significant risk factor of IR. Mainly started at T1DM or T2DM, diabetic nephropathy (DN) is a worldwide health problem, which is characterized by albuminuria and a decline of the glomerular filtration rate [26]. DN always correlates with chronic kidney disease and dysfunction of glomerular podocytes reveals a positive effect on the progression of DN. Many previous studies have indicated several altered regulatory factors and signalling pathways in DN, but the mechanism of DN remains elusive. Furthermore, various studies reported that diabetes could also increase the risk of progression of multiple cancers including the pancreas, esophagus, liver, colon, breast, and lung [27]. One study shows that diabetes could promote cell proliferation, invasion and metastasis of breast cancer in mice, and hyperglycemia contributes to cancer recurrence [28]. Meanwhile, high glucose level promotes tumour progression such as tumorigenesis, cell proliferation, anti-apoptosis, cell migration, cell invasiveness and drug resistance [29].

In the current review, we summarized the role of Cdc42 in insulin secretion, as well as the relationship between Cdc42 and diabetes-associated diseases, including IR, DN, and cancer. Furthermore, we hope to provide a theoretical basis for the design of more efficient anti-diabetic drugs in the treatment of diabetes and complications associated with DM.

## 2. Cdc42 and Insulin Secretion

**Figure 1:**
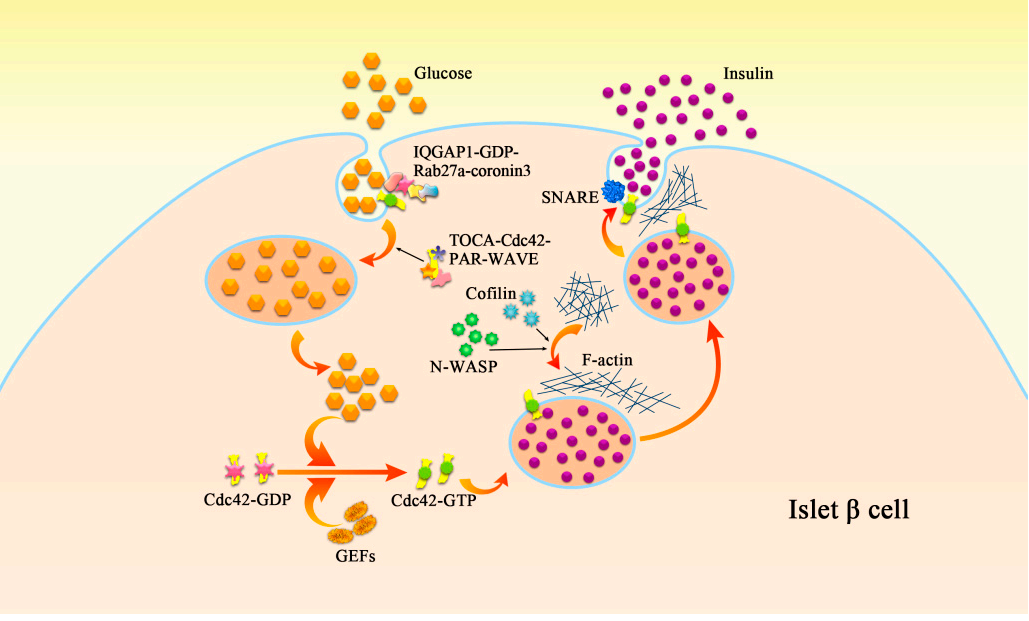
The mechanism of glucose-induced insulin secretion in islet β cell. GEFs exchange Cdc42-GDP (inactive) into Cdc42-GTP (active) which can be transported to the cell membrane, this process could be also promoted via glucose. Then activated Cdc42 is localized with insulin secretory granules and subsequently induces mobilization of insulin granules moving to the plasma membrane via rearrangement of F-actin, which could be rearranged via N-WASP and Cofilin as well. Positioning and fusion between granules and cell membrane are regulated by SNARE on cell membrane, and also by the F-actin rearranged by Cdc42. Glucose stimulates exocytosis as well as endocytosis of secretory membrane. Cdc42 is involved in the formation of a complex among IQGAP1, GDP-bound Rab27a, and coronin 3. This complex is essential for endocytosis of the insulin secretory membrane. Besides, a TOCA/Cdc42/PAR/WAVE complex contributes to endocytosis and is required for retrograde endocytic recycling.

GSIS refers to the process of releasing insulin and maintaining the balance of glucose in islet β cells under the stimulation of glucose [30]. Changes in many molecular mechanisms are involved in this process and lead to insulin secretion disorders. Among these, Cdc42 affects insulin secretion by regulating granule fusion, exocytosis and cytoskeletal rearrangement (Figure 1). Immunological and confocal microscopy observations confirmed the presence of Cdc42 in cloned oocytes, the islet of normal mice and human.

### 2.1. Cdc42 and β Cell Proliferation

Cdc42 is widely accepted as a pivotal mediator of cell proliferation by regulating cell cycle, and β cell proliferation depends on its positive effects as well. One study showed that up-regulation of miR-330-3p reduces expression of Cdc42 and E2F1 leading to impaired β cell proliferation via transferring from the plasma membrane to the cytoplasm of β cells in gestational diabetes mellitus (GDM) [31]. As downstream factors of Cdc42, p21-activated kinase 1(PAK1) is an essential promoter of cell proliferation. Study showed that PAK1 decreased 80% in T2DM, and subsequently both islet morphology and β cell mass, which were correlated with β cell function, were impaired under HG conditions [32]. On the contrary, another downstream factor of Cdc42, CyclinD1, which contributes to the proliferation of β cells, exhibits higher levels in islets of T2DM patients compared to healthy people [33]. This mechanism may slow down the progression of diabetes.

### 2.2. Cdc42 and Insulin Granule Mobilization

Second-phase insulin secretion requires the continual mobilization of insulin granules from synthetic parts to the plasma membrane, and this process mainly relies on filamentous actin (F-actin) remodelling regulated by Cdc42 and its downstream factors [34]. Currently, many studies have confirmed that Cdc42 activated PAK1 induces F-actin remodelling and insulin granules moving to the cell membrane via rapidly accelerated fibrosarcoma-1 (Raf-1)/mitogen-activated protein kinase (MEK)/extracellula regulated protein kinases (ERK) signalling pathway [35,36]. This may explain how Cdc42 promotes insulin particles moving to the cell membrane. In β cells, the specific interaction between vesicle-SNARE (v-SNAR) and syntaxin-1, subtypes of target-SNARE (t-SNARE) and synaptosome-associated protein 25 (SNAP-25) make vesicle close to target cell membrane and fuse [37]. At the same time, the downstream factors of Cdc42 could also regulate F-actin, such as neuronal Wiskott-Aldrich syndrome protein (N-WASP) and Cofilin. N-WASP binds Cdc42 to actin via the actin-related proteins 2/3 (Arp2/3) complex, and the interaction activated by Cdc42 between N-WASP and Arp2/3 provides a necessary condition for GSIS [23].

### 2.3. Cdc42 and the Exocytosis and Endocytosis of Insulin Granules

GSIS relies on the exocytosis of β cells. The Rho family regulates cytoskeletal remodelling and the fusion event in exocytosis in pancreatic β cells [12]. Cdc42 is localized with insulin secretory granules [38], and glucose converts GDP-bound Cdc42 to its GTP-bound form, thus promotes insulin secretion through modulation of the cortical actin network [39]. The exocytosis of insulin granules requires the positioning and fusion between granules and a “release region” on cell membrane. The bacterial glutathione-transferase-Cdc42 fusion protein was used in the co-immunoprecipitation and transient transfection tests to confirm that Cdc42 is the upstream regulator of cytokines, such as Syntaxin [40]. Positioning and fusion between granules and cell membrane are regulated by soluble N-ethylmaleimide-sensitive protein receptor (SNARE) on cell membrane, and also by the filamentous actin (F-actin) rearranged by Cdc42 [37]. In MIN6 β cells, glucose could activate Cdc42-PAK1-Rac1 signaling pathway, which was shown to play a regulatory role in the process of insulin exocytosis and the second stage of GSIS [41]. It has been demonstrated that binding of active Cdc42 and Rac1 activates PAK1, and then this mechanism initiates GSIS. However, synapses of amphids defective (SAD-A) kinase could directly phosphorylate PAK1 to induce insulin exocytosis in â cells without Cdc42 or Rac1 [42].

Previous experiments indicated that secretory stimulators activate Cdc42 [43], which could subsequently promote insulin granule exocytosis via rearrangement of F-actin [41]. Sato et al. found that inhibition of Cdc42 had adverse effects on secretory granule (SG) recruitment at the cell periphery [44], while inhibition of phosphoinositide-3 kinase promoted SG recruitment at the cell periphery by Cdc42-dependent actin reorganization [45]. Contrary to previous observations [44], Bretou et al. showed that inhibiting Cdc42 has little effect on SG recruitment at the cell periphery and no significant impact on SG docking but severely impairs full fusion, suggesting that the main effects of Cdc42 on exocytosis takes place at a post-docking stage. Their results indicated that knocking out Cdc42 reduces the size of newborn holes, slows their expansion, and may facilitate their premature closure which is called “kiss-and-run” [46].

In pancreatic β cells, glucose stimulates exocytosis as well as endocytosis of secretory membrane, maintains intracellular volume and sustains another round of exocytosis [47,48]. Researches on pancreatic β cells showed that, Cdc42 regulated the interaction between IQ domain GTPase-activating protein 1 (IQGAP1) and GDP-bound Rab27a [49]. IQGAP1 was involved in the regulation of vesicle tethering in insulin secretion [50,51]. Besides, IQGAP1 was identified as a novel GDP-dependent effector of Rab27a, and Cdc42-induced activation of IQGAP1 regulates the glucose-induced redistribution of Rab27a and coronin 3. Involvement of Cdc42 in the formation of a complex among IQGAP1, GDP-bound Rab27a, and coronin 3 was demonstrated. This complex is essential for endocytosis of the insulin secretory membrane. Apart from insulin secretory membrane, many significant membrane proteins, including the insulin-stimulated glucose transporter 4 (GLUT4), require retrograde recycling [52,53]. The mammalian transducer of Cdc42 dependent actin assembly (TOCA) was initially identified as an effector of Cdc42 [54]. TOCA facilitates Cdc42 linking to the WASP-family verprolin homologous protein (WAVE) complex. Furthermore, a TOCA/Cdc42/PAR/WAVE functional module is required for retrograde endocytic recycling [55].

Cdc42 is not only related to the proliferation of pancreatic β cells but also involved in both pre-docking and post-docking stage by modulating the concentration of calcium, promoting the transport of insulin granules via actin remodeling, and regulating the SG recruitment and membrane tension to affect the exocytosis of insulin granules. All these roles of Cdc42 demonstrate the necessity of Cdc42 in insulin secretion (Table 1). Although Cdc42 signalling is a known requirement for insulin secretion to occur, how it initiates remains unknown. Endocytosis is irreplaceable progress of insulin secretion as well, and Cdc42 plays a significant role in the endocytosis of secretory membranes and some membrane proteins (e.g., GLUT4 and TOCA). Cdc42 advances the progress of endocytosis through activating downstream factors or modulating downstream proteins structure (Table 1). Only with fast and precise endocytosis can pancreatic β cells reach the demand of insulin secretion.

## 3. Cdc42 and Diabetes-Associated Diseases

### 3.1. Cdc42 and Insulin Resistance

Insulin resistance refers to the decline in efficiency of insulin to promote glucose uptake and use for various reasons, and then the compensatory hyperinsulinemia happens [56]. Moreover, the hyperinsulinemia promotes the resistance of insulin in reverse [57]. It is widely convinced that insulin resistance is the primary cause of T2DM [24]. Changes in insulin structure, insulin receptors, obesity, and long-term hyperglycemia may all lead to insulin resistance and then increase the susceptibility of diabetes [58,59]. Above all, it is worth noting that obesity is the leading cause of insulin resistance, especially central obesity [59].

#### 3.1.1. Cdc42 and Declining Second-Phase of Insulin Secretion of T2DM Patients

Many studies have demonstrated that PAKs are effectors of small GTPases [60,61,62,63,64]. Research on T2DM donors’ islets manifests that PAK-1 protein loss average to 80% compared with non-diabetics, implicating the role of PAK-1 in islet insulin signalling functions [65]. Based on the analytical results of PAK-1 activation from human and rat islets, Cdc42 abundance is essential for PAK-1 activation [65]. The second/sustained-phase of insulin secretion is impaired by PAK-1 depletion from clonal MIN6 β cells, owing to the crucial role for PAK-1 as a Cdc42 effector in mediating cytoskeletal remodelling to facilitate insulin granule mobilization to the plasma membrane for insulin release [41]. From the aspect of decline of insulin secretion, the reduction of Cdc42 suppresses the activity of PAK-1 and may promote insulin resistance.

#### 3.1.2. Cdc42 and Peripheral Insulin Resistance

##### Regulation of Cdc42 on Insulin Signaling in Adipose Tissue

A high-fat diet (HFD) is a risk factor of systemic and muscular insulin resistance, hyperinsulinemia, and fat accumulation in insulin target organs [66]. In particular, abdominal obesity and fat accumulation in various organs may induce insulin resistance and peripheral blood sugar intolerance [67,68]. This may be associated with increased activity of hormone-sensitive triglyceride lipase (HSL) in patients with abdominal obesity. An in vivo study showed that HSL increased fat decomposition, which in turn elevated level of circulating free fatty acids (FFA) that increased fat accumulation and impaired insulin signaling, leading to insulin resistance in liver and peripheral tissues [69]. In skeletal muscle and adipose tissue, glucose uptake is stimulated mainly by GLUT4 [70]. Most of the GLUT4 (>90%) is restored in the pool of GLUT4, so the glucose uptake stimulated by insulin is mainly through the recruitment of GLUT4 to the plasma membrane [71]. Phosphoinositide 3-kinase (PI3K)/protein kinase B (AKT) pathway and the casitas B-lineage lymphoma (Cbl)/Rho-related GTP-binding protein (TC10) pathway are known to be involved in the insulin-stimulated GLUT4 translocation [72]. Insulin-stimulated translocation of GLUT4 is widely reduced in most animal and cellular models [73]. Cdc42 interacting protein-4 (CIP4) is involved in regulating cytoskeleton, membrane trafficking via interacting with the GTP-bound Cdc42 and positively related to insulin signalling and GLUT4 translocation through interacting with TC10. The in vitro study using 3T3 adipocytes suggests that insulin-stimulated GLUT4 translocation requires CIP4 protein via its interaction with TC10. The exposure of rats to HFD resulted in decreases of CIP4 and TC10 mRNA expression levels in the adipose tissue of rats [74].

Similarly, a research in endothelin-1 (ET-1) has emphasized the use of ET-1 treatment resulted in heterologous desensitization of insulin signalling, defined as chronic ET-1-induced cellular insulin resistance. Insulin receptor and ET-1 can promote the phosphorylation of the G protein αq/11-subunit (Gαq/11) via activating G protein-coupled receptor kinase 2 (GRK2) and subsequently inhibits Cdc42. Besides, GRK2 has already been proved to function as a negative regulator of insulin action [75]. Besides, anti-GRK2 antibody rescued this inhibitory effect [76]. Decline of Cdc42 activity and activated GRK2 resulted in decreased activity of Gαq/11 and down-regulation of GLUT4 translocation, ended up in inhibited insulin-stimulated glucose transportation and insulin desensitization. Thus, Gαq/11 participates in a pathway of insulin signalling to glucose transport via Cdc42 and PI3K [77,78].

##### Regulation of Cdc42 on Insulin Sensitivity in Liver Tissue

It is known that the occurrence of insulin resistance in liver tissue relies on the complicated feedback from excessive insulin [79]. C-Jun N-terminal kinase (JNK) activity and the occurrence of insulin resistance can be increased by obesity via increasing ER stress in hepatocytes [80], only JNK1 has been demonstrated to play a crucial role in regulating insulin resistance that related to obesity [81]. P85 regulatory subunits of phosphoinositide 3-kinase (PI3K) is involved in regulating JNK [82], and act as an upstream factor of Cdc42 through mediating Cdc42- mitogen-activated protein kinase 4 (MKK4) pathway [83]. To be specific, the combination of p85 with activated forms of Cdc42 relies on the joint between functional SH2 domains in the C terminus of p85 subunits and an intact N terminus of Cdc42 [82]. Decreased levels of p85 subunits suppress insulin resistance induced by HFD [84]. Taken together, we can draw a conclusion that JNK may be a center of the pathobiology of insulin resistance in liver, and may affect insulin signalling through Cdc42-MKK4 pathway.

##### Different Regulation of Rac1 and Cdc42 in Skeleton Muscle

Cdc42 and F-actin remodelling are known to be indispensable in insulin-stimulated GLUT4 vesicle translocation in skeletal muscle cells as a mean to evoke clearance of excess blood glucose [78,85]. Differring from the precise role of Cdc42 in adipose tissue, there is no firm evidence for the participation of Cdc42 in insulin action in skeletal muscle, and it is very likely that Rac1 signals to PAK1 in skeleton muscles [86,87]. Nevertheless, constitutively activated Cdc42, activator of PAK1 as well, was not able to stimulate GLUT4 translocation the activation of PAK1, therefore PAK1 may not be sufficient to menifest the induction of glucose uptake [88]. Although Cdc42, PAK-1, and Rac1 are known to participate in numerous F-actin remodelling and secretory events, their ordered use in these events can vary substantially. However, some researchers concluded contrary results from researches in brain neuron cells. Their results suggested Rac1 as a negative regulator of neuronal glucose uptake through regulating PAK2 activity, in contrast to its role in skeletal muscle [89]. Tissue-specific isoform abundance and functional contributions of small GTPases might be responsible for the different results obtained.

### 3.2. Cdc42 and Diabetic Nephropathy

DN is a complication associated with diabetes, and is characterized by persistent albuminuria, progressive decline in glomerular filtration rate (GFR), hypertension and sclerosis. Pathological changes including excessive deposition of extracellular matrix (ECM), thicken glomerular and tubular basement membranes, and increased mesangial matrix lead to glomerular sclerosis and tubulointerstitial fibrosis [90]. Inflammatory cytokines and tumor necrosis factor-alpha (TNF-α) are related to the development and progression of DN [91]. In the case of DN, evidence below demonstrate that Cdc42 controls diverse cellular functions including cell morphology, migration, endocytosis and cell cycle progression. It is borne out that Cdc42 participates in the progress of DN, while we try to discover whether Cdc42 play a role as a promoter or a suppressor under particular conditions and in different parts of the kidney.

#### 3.2.1. Cdc42 and Podocyte Injuries

Among the components of the glomerulus, podocytes are terminally differentiated and highly specialized cells in the Bowman’s capsule in the kidney that wrap around the capillaries of the glomerulus [92]. Podocytes play essential roles in the progression of DN and affect cytoskeletal actin dynamics [93]. Structurally, podocytes form a cell body through primary and secondary foot processes. The adjacent foot processes are connected by slit diaphragm proteins [94]. Injury to the function and structure of podocytes can induce apoptosis of podocytes in vivo or in vitro exposing to HG conditions, which can reduce the number of podocytes and damage normal cell morphology at the same time, leading to glomerulosclerosis and eventually a large amount of proteinuria [95,96,97]. In recent decades, many researchers consider Cdc42 as one of the factors which promote DN, especially takes part in podocyte injuries via the disturbed function of Cdc42 in podocyte cytoskeleton.

A wide range of receptors and enzymes contribute to the formation of injuries in podocytes through regulation of Cdc42. However, till now, part of the mechanism is still unexplored. For example, transforming growth factor-β1 (TGF-β1) is closely related to Cdc42 in DN. Recently, studies showed that podocytes exposed to angiotensin II (Ang II) or TGF-β1 showed up a substantial cytoskeletal rearrangement and significant loss of arch F-actin fibres in podocytes through increasing the activity of Cdc42, and probably led to the instability of cytoskeleton in podocytes [98]. Some results manifested that Cdc42 is related to the destabilization of the actin cytoskeleton of kidney podocyte, and probable pathways. Latest research showed that down-regulation of Slit-Robo GTP activating protein 2a in podocytes would reduce the binding of Cdc42 with SLIT-ROBO ρGTPase-activating protein 2a (SRGAP2a), suggesting that the role of SRGAP2a in stabilizing podocyte cytoskeleton is likely through its interaction with Cdc42 [99]. Similarly, PI3K regulates the activation of Cdc42 as well. Streptozotocin-induced proteinuric renal disease rats after treatment with wortmannin, a specific inhibitor of PI3Ks, showing reno-protective effects of wortmannin and restored Cdc42 in podocytes [100]. On the contrary, phosphatase and tensin homolog (PTEN) degrades phosphatidylinositol (3,4,5)-triphosphate (PIP3) into phosphatidylinositol 4,5-bisphosphate (PIP2), and this mechanism opposes the actions of PI3K [101]. Down-regulation of PTEN increases fibroblast motility also through stimulation of Rac1 and Cdc42 activity [102]. In podocytes, there is evidence that PIP3 induces actin polymerization by acting on signalling complexes mediated by PI3K [103].

Apart from actin cytoskeleton instability and rearrangement, some scientists suggest that Cdc42 affects the morphology and quantity of filopodia, also on cell migration. Ser71 phosphorylation of Rac1/Cdc42 increases filopodial structures, cell motility, and migration [104]. Ichii et al. observed an increase in phosphorylated Rac1/Cdc42 after indoxyl sulfate exposure in mouse podocytes results in increased cytoskeletal dynamics and decreased adhesion of podocytes [105]. On the contrary, Shen et al. found increases in the number and length of filopodia in podocytes, and demonstrated that activated *N*-methyl-d-aspartate receptors (NMDARs) played a vital role in DN by reducing Cdc42-GTP activation [106]. The elevated activation of Cdc42 results in a larger surface area and a reduced migration in NR1-sh podocytes [106]. According to Ichii and Shen’s studies, they respectively drew contrary conclusions on the role of Cdc42 in podocytes migration. This result may be due to the different technique of experiments and experimental design. Besides, other researches manifested that under the condition of hyperglycemia, remarkable oxidative stress response occurred in patients with diabetes. HG stimulation can induce the formation of radical oxygen species (ROS) in podocytes, leading to the apoptosis of podocyte [107]. In rat adipose-derived mesenchymal stem cells, the Cdc42 inhibitor decreased the levels of ROS, F-actin, but the activity of the ERK1/2 and JNK signalling pathways that were all elevated in these cells. However, the connection between Cdc42 and ROS in kidney podocytes is still elusive, with further experiments required.

Cdc42 in these cases acts as an anchor to maintain the shape and stability of the cell edge to enhance adhesion. Thus, it is plausible for us to speculate that up-regulation of expression or activation of Cdc42 may result in the decreased stabilization and rearrangement of the cytoskeleton, even decline adhesion of podocyte foot processes in vivo. These pathologic changes above may, in turn, promote the rise in proteinuria [108].

#### 3.2.2. Cdc42 and Mesangial Cell Hypertension and Disrupted Directionality of Migration

Apart from podocyte, the glomerular mesangial cell also plays an essential role in DN as well. DN is also characterized by glomerular mesangial cell hypertrophy. *gene 33* (also called mitogen-inducible gene-6, *mig-6*) is an immediate early gene that is rapidly induced by a heterologous array of mitogenic and stressful stimuli [109]. Transcription of *gene 33* is known to occur in response to insulin, growth factors, and some other stresses [110]. Expression of active high-sensitivity Cdc42 (Cdc42 Hs) promotes hypertrophy via activating the stress-activated protein kinase (SAPK) and p38 pathways, while the functional significance of the Gene 33/Cdc42 interaction and recruiting of SAPKs by Cdc42Hs remains unclear, therefore it is plausible to speculate that hypertrophy may occur due to sustained Gene 33-dependent SAPK pathway activation [109].

Other than hypertrophy, similar to podocytes, cytoskeleton of the glomerular mesangial cell may be disturbed as well. Cdc42 has been shown to play a role in cell motility and migration [111]. Cdc42 is active towards the front end of migrating cells; inhibition or global activation of Cdc42 results in disrupted directionality of migration [112]. In human mesangial cells with co-treatment of TGF-β and connective tissue growth factor (CCN2/CTGF), showed an increase in Cdc42 activation and Cdc42 effector kinases Pak1/2, but a decrease in migration, which increased in treatment with a single factor on the contrary [113,114].

#### 3.2.3. Cdc42 and Glomerulosclerosis

Recent studies have indicated that in vivo podocyte-specific deletion of Cdc42 leads to congenital nephrotic syndrome and glomerulosclerosis [115]. MicroRNAs are dysregulated in diabetic nephropathy as well, but the identification of specific microRNAs involved remains insufficient [116]. Some Ras signalling-related genes (e.g., Cdc42 and Rap1a/b) decreased significantly in miR-25 antagomir-treated mice. It is also further demonstrated that the overexpression of miR-25 in diabetic mice could even reverse these gene alterations [117].

#### 3.2.4. Cdc42 and Tubulointerstitial Fibrosis

The presence of progressive tubulointerstitial fibrosis implied an unalterable fate, linking to the final stage of renal failure. Epithelial-to-mesenchymal transition (EMT) plays a significant role in chronic kidney fibrosis as well as cancer progression [118]. During progressive tubulointerstitial fibrosis, renal tubular epithelial cells transform into α-smooth muscle actin (SMA)-expressing myofibroblasts via EMT. In LLC-PK1 cells, TGF-β1 regulates the SMA expression. Cdc42 activates SMA promoters. Kidneys of diabetic rats are depicted significant increase of SMA, suggesting that Cdc42 regulates EMT and renal fibrosis via SMA [119]. Similarly, Yu et al. studied the positive effect of TGF-β1 on CIP4 via PI3K/AKT pathway in renal tubular EMT. CIP4 was also increased in an experiment of renal interstitial fibrosis [120]. CIP4 can directly promote the phosphorylation of multiple signalling molecules, owing to its tyrosine protein kinase activity [121]. In return, CIP4 may further participate in TGF-β1 induced EMT [122]. Therefore, it is plausible to speculate that the microenvironment changes, mediated by higher expression of signalling molecule TGF-β1, result in renal tubular EMT and more severe tubular interstitial fibrosis [123,124].

Cdc42 plays an indispensable role in pathological changes of DN. The most noteworthy part is in podocyte injuries. On account of the ability to control cytoskeleton, dysregulation of Cdc42 is related to the morphology of podocyte filopodia and process. In podocytes, receptors and enzymes involved (e.g., TGF-β, SRGAP2a, and PTEN) are the primary influence factors of Cdc42. Apart from that, Cdc42 participates in the regulation of hypertrophy and migratory capacity of glomerular mesangial cells. Furthermore, Cdc42 might take part in the glomerulosclerosis tubular interstitial fibrosis. Some of the upstream factors and the level of Cdc42 activation may be used as a specific marker for DN progress, providing new methods for clinical treatments. However, some of the mechanisms remain unclear and require further experimental exploration.

### 3.3. Cdc42 and Cancer Under Hyperglycemia

Results from earlier studies demonstrate a strong and consistent association between diabetes and an increasing risk of various types of cancer and the mortality rate of cancer patients [125,126]. The relationship of diabetes and cancers has attracted more attention in recent decades due to the sharp rise in the number of people with diabetes worldwide and current findings, suggesting that some anti-diabetes treatments appear to reduce cancer risk [126,127]. As demonstrated above, Cdc42 involves in the pathomechanism of diabetes, here we try to figure out whether Cdc42 relates to cancers under hyperglycemic condition.

#### 3.3.1. Cdc42 and Cancer Cell Growth and Survival under Hyperglycemia

HG conditions significantly increases the proliferation of breast cancer cells (e.g., MDA-MB-231, SKBR3 and MCF-7 cells) compared to low glucose condition [126]. The promotion of cancer cell proliferation may be due to activating the mitogenic signalling by modulating epidermal growth factor receptor (EGFR) activation through Cbl and GTPases Cdc42 [128]. In detail, Cbl proteins catalyze the degradation of EGFR [129]. Cdc42 blocks this course and influences EGFR activation through or independent of Cool/βPix proteins, which are GEFs for Rho family GTPase [128,130]. Increased glucose metabolism is one of the characteristics of proliferative cancer cells. In human acute myeloid leukemia (AML), transformation by mutant *CBL* depends on functional expression of Cdc42 and increased glucose metabolism. Phosphorylation on *CBL* increases the recruitment of nucleotide exchange factors [131], including a guanine nucleotide exchange factor for Rac and Cdc42 [132]. Upregulated Vav1 increases Cdc42 activation and cell growth. Because small GTPases are target proteins of these nucleotide exchange factors and have been implicated in contributing to cell growth [133].

Apart from hyperglycemia, hyperinsulinemia can also be found in diabetes patients. Insulin shows pleiotropic effect. It not only regulates the metabolic processes of many cells but can also regulate cell growth and differentiation [134]. Human insulin increases the level of miR-29a in ER-positive breast cancer cells (e.g., MCF-7 cell and T47D cell) [135], and the expression of miR-29a was elevated under hyperglycemic condition, altogether triggering ERK phosphorylation (mainly contributing to the proliferation), Cdc42 downregulation and MAPK pathway inactivation [135]. The effect of activating ERK outstrips the impact of suppressing MAPK pathway and ultimately promotes breast cancer cell proliferation. This mechanism may explain the proliferative behaviour caused by high insulin in MCF-7 cells and T47D cells.

Cdc42 not only participates in cancer cell growth but also survival under particular circumstances. Recently, involvement of Cdc42 in Burkitt lymphoma cells resistance to ascorbate-induced cytotoxicity was discovered. Evidence showed that Cdc42 promoted survival of Burkitt lymphoma cells through regulating major histocompatibility complex (MHC) and myosin light chain (MLC) [136].

#### 3.3.2. Cdc42 and Cancer Cell Invasion under Hyperglycemia

Despite the abundant study of Cdc42 in cancer cell proliferation, surprisingly little is known about the relationship between Cdc42 and cancer cell invasion under HG condition. According to Warburg’s effect, cancer cells need high glucose levels for their metabolism. In these cells ATP is produced during glycolysis, therefore higher amounts of glucose are necessary. On this basis, some key factors, such as glucose transport carriers, glycolytic and glutamine pathways are suggested playing a significant role in this change [137]. It is demonstrated that hyperglycemia promotes invasion and cancer stem cell (CSC) activity through miR-424-Cdc42-prdm14 signalling axis in MDA-MB-231 cells [138]. In hyperglycemia, impaired inhibitory regulation of miR-424 on Cdc42 leads to the activation of prdm14 (PR-domain containing 14) which maintains pluripotency and represses differentiation [138,139].

#### 3.3.3. Cdc42 and Cancer Cell Migration and Metastasis under Hyperglycemia

Diabetes can be generally characterized by hyperglycemia. However, in long-term treatment of diabetes, hyperglycemia is a relatively common reaction and one of the emergencies especially in the use of insulin, which acts as hypoglycemic factor [140]. There are relatively few studies in the area of cancer under hypoglycemia. For example, a recent study found that, in human colon cancers, MYC-nick promotes migration and survival of DLD1 and HCT116 in response to withdrawal of glucose. At great length, MYC-nick promoted a sustained activation of Cdc42 and increased fascin expression to induce filopodia formation and to drive migration and metastasis in DLD1 and HCT116 [141].

Taking as a whole, evidence indicates that activation of Cdc42 participates in the progression of cancer cell proliferation, survival, invasion, and migration under hyperglycemic condition (Table 2). However, the role of Cdc42 in cancer-related to diabetes or high glucose condition is still partly unclear and remains to be discovered with further study.

## 4. Conclusions and Future Perspectives

**Figure 2:**
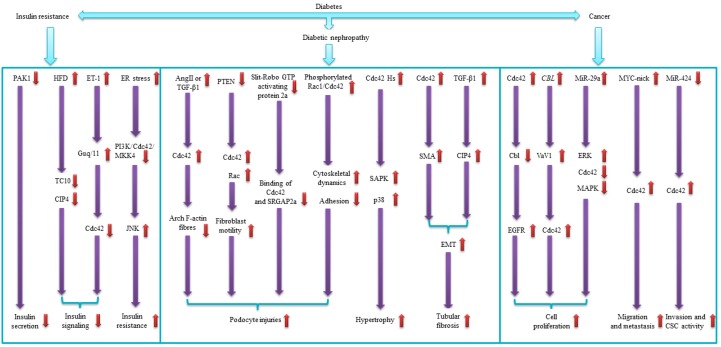
Cdc42 and diabetes associated diseases. This figure depicts the signaling pathways that Cdc42 may impact diabetes associated diseases including insulin resistance, diabetic nephropathy and diabetic cancer. Arrows in purple point to downstream factors, and arrows in red refer to regulation of them. The up (down) arrow in red indicates that the corresponding factor is up-regulated (down-regulated).

Diabetes is a global public health concern. Diabetes is characterized by multiple mutagenic events that affect the secretion and sensitivity of insulin. Moreover, diabetes is known to be associated with various diseases in different tissues and organs, including kidney, skeleton muscle, eyes, lung, heart, liver, and even brain [142,143,144,145,146,147,148]. The protein Cdc42 is a member of the Rho family of small GTPases. Cdc42 controls signal-transduction pathways that lead to rearrangement of the cell cytoskeleton, cell differentiation and cell proliferation by binding to downstream effector proteins [149,150,151]. These functions of Cdc42 are associated with diabetes (Table 1). In this review, we suggest that Cdc42 also regulates diabetes-associated diseases such as IR, DN and different types of cancers (Table 2, Figure 2). IR is one of the well-studied features of metabolic syndrome that may be influenced by many risk factors [152]. The chronic inflammation in the adipose tissue or liver, mainly during obesity, has been linked to insulin sensitivity [153]. Furthermore, adipose tissue insulin resistance is one of the pathophysiological components of type 2 diabetes [154]. On the contrary, correlation of Cdc42 and IR in some tissues (such as skeleton muscles and neuron cells) remains unclear and shows significant variety in our review [86,87]. DN is one of the common compilations of diabetes. Studies have demonstrated the relationship between diabetes and kidney disease secondary to it [155,156]. Inflammatory cytokines and tumour necrosis factor-alpha is related to the development and progression of DN [91]. In this review, we show an intimate association between Cdc42 and renal damages (Table 2). Cdc42 is implicated in multiple human cancers and epithelial to mesenchymal transition, via regulating various signalling pathways [157,158,159]. There is now a sufficient level of evidence for the association between diabetes and cancers as well [160,161]. Besides, T2DM may act as a predictor of survival among breast cancer patients [162]. Despite the fact that Cdc42 is highly discussed in diabetes as well as in cancers, researches aiming at Cdc42 and cancers under HG condition were limited. Therefore, we try to figure out whether there is a connection between diabetes and cancer by Cdc42 through summarizing the evidence we collected. However, a large portion of the mechanisms remains unclear and may require further experimental exploration.

In the current review, we summarized new evidence of the role of Cdc42 in diabetes, IR, DN, and cancer, in the hope of providing the basis for clinical diagnosis and therapy of DM and other diabetes-associated diseases. We demonstrate the interaction among Cdc42, diabetes and diabetes-associated disease (Table 1 and Table 2).

⮚ Cdc42 can affect insulin secretion via (a) promoting the proliferation of β cells by regulating PAK-1 and CyclinD1; (b) controlling the insulin granule mobilization and the exocytosis of insulin granules through signaling pathway such as: Raf-1/MEK/ERK, TOCA/Cdc42/PAR/WAVE and Cdc42-PAK-1-Rac, and proteins such as N-WASP and Arp2/3; (c) separation and binding of the t-SNARE all related to activated Cdc42; (d) SG recruitment.
Cdc42 impairs the insulin secretion and promotes IR via suppressing the activity of PAK-1. Cdc42 can affect IR in adipose tissue by (a) interaction of CIP4 with TC10; (b) Gαq/11 participating in Cdc42 and PI3K to mediate insulin signalling to glucose transport. Cdc42 can affect IR in the liver by being activated by PI3K and regulating insulin sensitivity through MKK4. Cdc42 suppresses IR in skeleton muscles by regulating F-actin and cytoskeleton to transport GLUT4.
Cdc42 regulates the pathogenesis of DN by (a) promoting podocyte injury through interacting with some receptors and enzymes (such as TGF-β, SRGAP2a, PI3K, PTEN, and NMDARs); (b) protecting podocyte via restoring in SD rats; (c) regulating hypertrophy and migratory capacity of glomerular mesangial cell; (d) participating in glomerulosclerosis tubular interstitial fibrosis.
Cdc42 regulates cancer cell proliferation under HG condition by (a) activating Cbl and EGFR in MDA-MB-231, SKBR3, MCF-7; (b) interacting with Vav1 in Baf3 or with miR-29a in MCF-7. Cdc42 regulates cancer cell invasion in hyperglycemia by promoting CSC activity through miR-424-Cdc42-prdm14 signalling axis in MDA-MB-231. Cdc42 regulates cancer cell metastasis by increasing fascin expression to induce filopodia formation in DLD1 and HCT116.

Overall, large and growing body of literature has investigated a causal relationship between activated Cdc42 and the physiological function of the pancreatic β cells. It has been conclusively shown that Cdc42 acts as a promoter in the secretion of insulin. Apart from that, probably due to the tissue specificity, Cdc42 plays different or even opposite roles in some diabetes-associated diseases. Thus, substantial opportunity exists for further studies to define how Cdc42 participates in progress associated with diabetes (such as IR in skeleton muscle, the damage of epithelia of renal tubules in kidney, or the metastasis and autophagy of cancer cells under HG condition). Other than diseases we discussed above, Cdc42 is linked to diabetic cardiomyopathy as well, and acts as a critical downstream factor of miR-30c [163]. Furthermore, we suggest detecting the expression level and activation of Cdc42 in β cell and other tissues will be of significant diagnostic and potential therapeutic value, for Cdc42 is considered linked to the pathogenesis, progression, and complications of diabetes.

For the moment, a systematic understanding of how Cdc42 contributes to therapies of diabetes is still lacking. It is, therefore, essential to further characterize the mechanisms of regulated Cdc42 function in diabetes pathogenesis and identify targeted approaches that synergize or supplement traditional therapies. Insulin has been widely used to treat diabetes in the past many years, but insulin increases the risk of overall, pancreatic, and colorectal cancer [164]. Up to now, there is a notable paucity of high-quality research focusing specifically on drugs treating diabetes. TNBC is manifested as ER-negative, progesterone receptor (PR)-negative, and HER2-negative, and lacks specific clinical therapeutic guidelines [165]. Luckily, the antidiabetic drug metformin, which has been reported to inhibit breast cancer cell proliferation and migration by significantly downregulating Cdc42 expression, acts as a potential anti-cancer therapy to treat TNBC [166,167]. When treated with metformin, an increased level of AMP activates AMP-activated protein kinase (AMPK), which inhibits the mammalian target of rapamycin (mTOR) expression to suppress tumor progression, metformin exhibits anti-cancer activity via this AMPK signaling pathway [168]. However, metformin-mediated Cdc42 downregulation does not require this typical AMPK signaling pathway. Conversely, AMPK signaling pathway upregulates Cdc42 expression [169]. Downregulation of Cdc42, induced by metformin, is partially due to transcription factors such as deoxynucleotidyltransferase terminal-interacting protein 2 (DNTTIP2), transcription elongation factor B polypeptide 2 (TCEB2), and 14-3-3 protein beta/alpha (YWHAB). [169]. In recent years, many researchers concentrate on Chines medical therapy. Lately, a study on the effect of Chinese herb on T2DM demonstrated that Yi-Qi-Yang-Yin-Hua-Tan-Qu-Yu (YQYYHTQY) recipe showed therapy effect on T2DM. Cdc42 and RhoA proteins were the therapy targets of YQYYHTQY recipe [170]. The growing field of Cdc42 thus seems poised to provide new insights into both pathological basis and drug designing. Strategies target on Cdc42 and its downstream factors activities during the progression of diabetes, and diabetes-associated diseases could have therapeutic potential. As such, further analysis of the relationship between Cdc42 and diabetes diagnosis, targeted treatment, prognosis, and therapeutic response may uncover important new roles of Cdc42, with possible clinical value. Through this review, we hope to provide a theoretical basis for Cdc42 to be designed as drug-target, and treatment targeting Cdc42 may be one of the effective treatments for patients with diabetes and associated diseases.

## Figures and Tables

**Table 1:** Cdc42 and insulin secretion.

Processes	Cell Lines/Tissues	Signalling Pathways	Promoter/Suppressor	References
Pancreatic β cell proliferation	MIN6	Yes/Cav-1/Cdc42/PAK-1	Promoter	[35]
	blood of GDM patients	miR-330p/Cdc42	Promoter	[31]
	INS 832/13	Cdc42/PAK-1	Promoter	[32]
	male C57BL/6J mice	Cdc42/cyclinD1	Promoter	[33]
Granules mobilization	MIN6-K8 β cell	Cdc42/N-WASP/Arp2/3	Promoter	[23]
	MIN6	Cdc42/PAK-1/MEK/ERK	Promoter	[36]
Secretory membrane exocytosis	MIN6	Cdc42/PAK-1/Rac1	Promoter	[41]
	islets of db/db mice	GGPPS/GGPP/GGTase1/Cdc42	Promoter	[21]
Secretory membrane endocytosis	MIN6	Cdc42/IQGAP1	Promoter	[49]
	C.elegans intestine	TOCA/Cdc42/PAR/WAVE	Promoter	[55]

**Abbreviations:** PAK-1: p21-activated kinase 1; YES: protein of the Src family; GDM: gestational diabetes mellitus; N-WASP: neuronal Wiskott-Aldrich syndrome protein; Arp2/3: actin-relatedprotein2/3; MEK: mitogen-activated protein kinase; ERK: extracellular regulated protein kinases; GGPPS: geranylgeranyl pyrophosphate synthase; GGPP: geranylgeranyl pyrophosphate; GGTases-1: geranylgeranyltransferase-1; IQGAP1: IQ domain GTPase-activating protein 1; TOCA: transducer of Cdc42 dependent actin assembly; PAR: partitioning defective; WAVE: WASP-family verprolin homologous protein.

**Table 2:** Cdc42 and diabetes-associated disease.

Diabetes-Associated Diseases		Cell Lines/Tissues	Signalling Pathways	Promoter/Suppressor	References
IR	Insulin secretion	MIN6, human islets	Cdc42/PAK-1	Suppressor	[41,65]
	Adipose tissue	male Sprague-Dawley rats	CAP/Cbl/TC10/CIP4	Suppressor	[74]
	Liver	3T3-L1 adipocytes	ET-1/Cdc42/Gαq/11s	Suppressor	[76]
		CV1, HeLa	PI3K/Cdc42/MKK4/JNK	Promotor	[83]
DN	Podocytes injury	immortalized mouse podocytes	Ang II or TGF-β1/Cdc42	Promotor	[98]
		podocytes of DN patients specimens	Binding of Cdc42 with SRGAP2a	Promotor	[99]
		SD rats	Wortmannin/PI3K/Cdc42	Suppressor	[100]
		male Sprague-Dawley rats	Nephrin/PTEN/PIP3/Cdc42	Promotor	[103]
	Podocytes migration	C57BL/6, FVB/N mice	phosphorylated Rac1/Cdc42	Promotor	[105]
		C57BL/6J, db/db mice, db/dm mice	NMDARs/Cdc42	Suppressor	[106]
	Mesangial cell injury	human embryonic kidney 293 cells, A549, rat renal mesangial cells	Gene 33/Cdc42/SAPK/p38	Promotor	[109]
		HMCs	TGF-β, CCN2/Cdc42/PAK	Suppressor	[113,114]
	Glomerulosclerosis Tubular fibrosis	db/db mice	miR-25/Cdc42	Suppressor	[115,117]
		LLC-PK1	Cdc42/SMA promoters	Promotor	[119]
		SD rats	TGF-β/PI3K/AKT/CIP4	Promotor	[122]
Cancer	Cancer cell growth and survival	MDA-MB-231, SKBR3, MCF-7	HG/Cdc42/Cbl/EGFR	Promotor	[128]
		Baf3	CBL/Vav1/Cdc42	Promotor	[131]
		MCF-7, T47D	miR-29a/Cdc42	Suppressor	[135]
		JLPs/JLPR Burkitt lymphoma cells	Cdc42/MHC, MLC	Promotor	[136]
	Cancer cell invasion	MDA-MB-231	miR-424-Cdc42-prdm14	Promotor	[138]
	Cancer cell metastasis	DLD1 and HCT116	MYC-nick/Cdc42	Promotor	[141]

**Abbreviations:** CAP: Cbl-associated protein; Cbl: casitas b-lineage lymphoma; Tc10: Rho-related GTP-binding protein; CIP4: Cdc42 interacting protein-4; ET-1: endothelin-1; Gαq/11: G protein αq/11-subunit; JNK: c-Jun N-terminal kinase; PI3K: phosphoinositide 3-kinase; MKK4: mitogen-activated protein kinase kinase 4; TGF-β1: transforming growth factor-β1; CCN2/CTGF: connective tissue growth factor; Ang II: angiotensin II; SRGAP2a: SLIT-ROBO ρGTPase-activating protein 2a; PTEN: phosphatase and tensin homolog; PIP3: phosphatidylinositol (3,4,5)-triphosphate; NMDARs: *N*-methyl-d-aspartate receptors; SAPK: stress-activated protein kinase; HMC: human mesangial cells; SMA: smooth muscle actin; HG: high glucose; EGFR: epidermal growth factor receptor; CBL: casitas B-lineage lymphoma; MHC: major histocompatibility complex; MLC: myosin light chain; prdm14: PR-domain containing 14.

## Abbreviations

AKT	Protein kinase B
AML	Human acute myeloid leukemia
Ang II	Angiotensin II
Arf	ADP-ribosylation
CAP	Cbl-associated protein
Cav-1	Caveolin-1
CBL	Casitas B-lineage lymphoma
CCN2/CTGF	Connective tissue growth factor
Cdc42	Cell division cycle 42
Cdc42 Hs	High-sensitivity Cdc42
CIP4	Cdc42 interacting protein-4
CSC	Cancer stem cell
CysLT1R	Cysteinyl-leukotrienes receptor 1
DM	Diabetes mellitus
DN	Diabetic nephropathy
ECM	Extracellular matrix
EGFR	Epidermal growth factor receptor
ERK	Extracellula regulated protein kinases
GDI	GDP-dissociation inhibitor
GDM	Gestational diabetes mellitus
GEF	Guanine nucleotide exchange factor
GFR	Glomerular filtration rate
GGPP	Geranylgeranyl pyrophosphate
GGPPS	Geranylgeranyl pyrophosphate synthase
GGTases-1	Geranylgeranyltransferase-1
GLUT	Glucose transporter
GRK2	G protein-coupled receptor kinase 2
GSIS	Glucose-stimulated induced insulin secretion
Gαq/11	G protein αq/11-subunit
HFD	High-fat diet
IQGAP1	IQ domain GTPase-activating protein 1
IR	Insulin resistance
JNK	C-Jun N-terminal kinase
MEK	Mitogen-activated protein kinase
MHC	Major histocompatibility complex
MKK4	Cdc42- mitogen-activated protein kinase 4
MLC	Myosin light chain
NMDARs	*N*-methyl-d-aspartate receptors
NOX4	NADPH oxidase 4
N-WASP	Neuronal Wiskott-Aldrich syndrome protein
PAK-1	p21-activated kinase 1
PI3K	Phosphoinositide 3-kinase
PIP2	Phosphatidylinositol 4,5-bisphosphate
PIP3	Phosphatidylinositol (3,4,5)-triphosphate
prdm14	PR-domain containing 14
PTEN	Phosphatase and tensin homolog
Raf-1	Rapidly accelerated fibrosarcoma-1
ROS	Radical oxygen species
SAD-A	Synapses of amphids defective
SAPK	Stress-activated protein kinase
SG	Secretory granule
SMA	α-smooth muscle actin
SNAR	Soluble N-ethylmaleimide-sensitive protein receptor
SRGAP2a	SLIT-ROBO ρGTPase-activating protein 2a
T1DM	Type 1 diabetes mellitus
T2DM	Type 2 diabetes mellitus
TC10	Rho-related GTP-binding protein
TGF-β1	Transforming growth factor-β1
TOCA	The transducer of Cdc42 dependent actin assembly
WAVE	WASP-family verprolin homologous protein
